# Plant cell cultures as food—aspects of sustainability and safety

**DOI:** 10.1007/s00299-020-02592-2

**Published:** 2020-09-06

**Authors:** Suvi T. Häkkinen, Heli Nygren, Liisa Nohynek, Riitta Puupponen-Pimiä, Raija-Liisa Heiniö, Natalia Maiorova, Heiko Rischer, Anneli Ritala

**Affiliations:** VTT Technical Research Centre of Finland, Tietotie 2, P.O. Box 1000, 02044 VTT Espoo, Finland

**Keywords:** Cellular agriculture, Dairy side stream, Food, Lactose, Plant cell cultures, Plant growth regulators

## Abstract

**Key message:**

**Sustainability and safety aspects of plant cell cultures as food are presented. Applicability of dairy side streams as carbon source and use of natural growth enhancers in cultivation are shown**.

**Abstract:**

Biotechnologically produced cellular products are currently emerging to replace and add into the portfolio of agriculturally derived commodities. Plant cell cultures used for food could supplement current food production. However, still many aspects need to be resolved before this new food concept can enter the market. Issues related to sustainability and safety for human consumption are relevant for both consumers and regulators. In this study, two plant cell cultures, deriving from arctic bramble (*Rubus arcticus*) and birch (*Betula pendula*), were cultivated using lactose-rich dairy side streams as alternative carbon sources to replace sucrose. Biomasses were comparable to those of original plant cell culture media when up to 83% and 75% of the original sucrose was replaced by these side streams for arctic bramble and birch cell cultures, respectively. Furthermore, nutritional composition or sensory properties were not compromised. Synthetic plant growth regulators were replaced by natural components, such as coconut water and IAA for several subculture cycles. Finally, it was shown that only trace amounts of free growth regulators are present in the cells at the harvesting point and assessment by freshwater crustaceans assay indicated that toxicity of the cells was not exceeding that of traditionally consumed bilberry fruit.

**Electronic supplementary material:**

The online version of this article (10.1007/s00299-020-02592-2) contains supplementary material, which is available to authorized users.

## Introduction

Novel, sustainably produced and nutritious food sources are needed to feed the growing human population. It is estimated that we will be 10 billion in 2050 and that the current food chain will not be efficient enough to provide nutritious food for everybody. Cellular agriculture, i.e., the utilisation of a wide variety of cell cultures for the production of agricultural commodities could be supplementing current production by farmed animals or crops (Davies and Deroles 2014; Rischer et al. 2020). Plant cell cultures have shown great potential for food purposes, with their relatively high protein, energy and fiber contents (Nordlund et al. 2018). Plant cell cultures can be established of any plant species and grown in containment independently of environmental factors, e.g., climate or seasonal variations. Due to cultivation in containment, plant cell cultures are devoid of field-borne pathogens such as viruses and other pests. From the sustainability perspective, the bioreactor grown plant cell culture biomass can be completely utilized or at least creation of new waste or side streams is minimized. Thus plant cell cultures offer an attractive option in the food production portfolio (for recent reviews see Eibl et al. 2018; Rischer et al. 2020).

Economics hindering broad use of plant cell cultures at large scale were recently investigated revealing that one of the important cost factors in plant cell culture technology is the culture medium. This study identified and optimized the most cost-relevant nutrients in the culture medium leading to reduction of the costs for biomass production up to 43–55% (Häkkinen et al. 2018). This was mainly achieved by altering the amounts of the most expensive minerals in the culture medium. However, the seventh most expensive culture medium component in Murashige and Skoog-based medium is sucrose, which is the most typical and sole carbon source in plant cell culture media. Sucrose is a component which affects the sustainability aspect of plant cell cultures, too, since sucrose originates from agricultural production and is as such a high energy source for human nutrition. Alternative carbon sources explored for plant cell cultivation in the past include monosaccharides and disaccharides as well as sugar alcohols, polysaccharides and organic acids, however, very limited success has been achieved (Wu and Ho 1999). Thus, there is continued interest in exploring alternative and food-grade carbon sources for heterotrophic plant cell cultivation. In this study we examined dairy side streams high in lactose, which currently have a rather modest recycling or re-use value and thus novel high added-value concepts are constantly looked for in the dairy industry. Dairy industry waste management is a significant issue, since without appropriate treatment, the effluents with high organic load and nutrients such as nitrogen and phosphorous pose serious environmental hazard (Rivas et al. 2011; Prazeres et al. 2012). The EU environmental regulation stipulates that these dairy side streams must be reused to a large degree and cannot be disposed off as waste (personal communication, Antti Heino). When used as plant media ingredients such side streams could on the other hand further fortify the nutritional and sensory value of plant cells as food. Therefore we carefully monitored these properties to evaluate their impact for this specific application area.

Plant cell and tissue cultures are cultivated on growth media often supplemented with one or two plant growth regulators. Auxins, cytokinins and gibberellins are typical growth regulator classes with functions in, e.g., cell division, cell cycle, germination and flowering (Davies 1995). Several synthetic growth regulators have been chemically made based on the first isolated natural growth regulators IAA (indole acetic acid), zeatin and gibberellic acid. Although natural growth regulators found, e.g., in fruits and vegetables are considered safe to consume at native levels, the synthetic growth regulators pose different risks. They are used as pesticides in agriculture and allowed residual levels in treated products are legally restricted. In plant cell culture media growth regulators are indispensable but they are added in physiological amounts, i.e., trace levels. Nevertheless, until now it has been unclear which amount of the initially added growth regulators are still present at the end of the cultivation cycle. In this study we measured the growth regulator concentration at the time of biomass harvesting and evaluated the toxicity of the harvested plant cells using a crustacean toxicity screening test for freshwater. This information is crucial for novel food compliance of plant cell cultures. In addition it was attempted to substitute synthetic growth regulators altogether in the propagation of plant cell cultures as food.

## Materials and methods

### Plant cell culture material

Lactose-containing dairy side-streams were obtained fresh from Valio Oy, Finland and stored at + 4 °C until the usage. Four different side-streams were utilized: Lactose fraction (LS-1), UF-milk permeate (LS-2), Acid whey (LS-3), UF-whey permeate (LS-4). The composition of side-streams is shown in Supplementary Table S1. Various amounts of side-streams or other additives (coconut water (Sigma, C5915), IAA (Sigma, I1250), casein hydrolysate (Sigma Hy-Case SF from Bovine), yeast extract (Amresco J850)) were added to the original culture medium before autoclaving. Amounts of added lactose-containing side-streams were calculated based on total carbohydrate (w/v) in the original medium, assuming equal weight ratios for sucrose and lactose. Plant cell cultures of arctic bramble (*Rubus arcticus*, VTTCC P120089) were established from sterilized plant explants and maintained as described (Nohynek et al. 2014). Embryogenic cell line of silver birch (*Betula pendula* Roth) was originally established from leaves (Kurtén et al. 1990). The cell line was cryopreserved and stored in liquid nitrogen in VTT cell culture collection (https://culturecollection.vtt.fi). For this study the embryogenic cell line E1 (VTTCC P120047) was taken out of cryo-storage, and callus culture was established showing high viability and embryogenic capacity. The callus culture was maintained on solidified N7 medium (Simola 1985) supplemented with the plant growth regulators 2,4-D (2,4-dichlorophenoxyacetic acid 2,4-D, Sigma) (2 mg/l) and kinetin (Sigma) (0.5 mg/l). Subculturing interval was 3 weeks. Following cultivation parameters were used: T =  + 25 °C, photoperiod = 16 h:8 h (light:dark), irradiation about 50 µmol/m^2^sec. Suspension culture established from the callus culture was subcultured to fresh medium every 10 days. About 3 g FW of the cells were transferred to fresh medium (75 ml in 250 ml Erlenmeyer flasks). The cultivation parameters were the same as for callus culture described above, and agitation of the flasks was 110 rpm. The suspension was first sieved using 1.0 mm metal sieve to obtain homogeneous suspension culture. Cultures were maintained in liquid cultures in 250 ml flasks, with a working volume of 50 ml. Cells were pre-cultivated for 8 days and transferred then to each side-stream containing medium. Experiments were performed in 20 ml working volume in 100 ml Erlenmeyer flasks, unless otherwise indicated. After 8-day cultivation, samples were collected and biomass determined. Results are expressed as mean (± SD) of three biological replicates, unless otherwise indicated.

### Evaluation of sensory characteristics of plant cell cultures

Sensory evaluations were performed as described in our previous publication (Nordlund et al. 2018). The descriptive panel consisted of ten trained assessors with proven skills. All sensory work of the plant cell cultures was carried out at the sensory laboratory of VTT Technical Research Centre of Finland Ltd., which fulfils the requirements of the ISO standards (ISO 6658, 2017 and ISO 8589, 2007). All assessors on the internal sensory panel have passed the basic taste test, the odour test and the colour vision test. They have been trained in sensory methods at numerous sessions over several years, and their evaluation ability is routinely checked. The panel was particularly familiarized with the sensory descriptors and the attribute intensities of various plant-based materials, including berries, in several sessions prior the evaluations. In accordance with EU General Data Protection Regulation GDPR (2016/679), necessary individual information of the members of the panel is collected in the data protection registry, and the panelists have also given their consent for this. The protocol for performing the sensory evaluation has been accepted by the Ethical Committee of VTT (Supplementary Appendix 1).

The samples subjected to sensory trials were (A) Arctic bramble cell culture cultivated in standard cultivation medium (100% sucrose) and in medium with 25% sucrose + 125% LS-2, and (B) Birch cell culture cultivated in standard cultivation medium and in 25% sucrose + 75% LS-3; 50% sucrose + 50% LS-4 and 50% sucrose + 50% LS-2. Arctic bramble and birch cell cultures were assessed separately. The method in sensory profiling was descriptive analysis (Lawless and Heymann 2010). The attributes were carefully defined and described verbally together with the panelists, and the ends of the intensity scales of the attributes were anchored verbally. The attribute intensities (0 – 10) were rated on continuous graphical intensity scales, verbally anchored from both ends, where 0 = attribute not existing, 10 = attribute very clear. The evaluated attributes of the arctic bramble and birch cell cultures were odour freshness, coarseness of texture, sweetness, sourness, and intensity of possible off-flavour. The samples were coded with three-digit numbers and served to the assessors in random order as such from odourless disposables covered by a lid accompanied with a spoon in one session. The scores were recorded and collected using a computerized Compusense Five data system, Ver. 5.6 (Compusense, Guelph, Canada). The means of the raw data obtained from the sensory evaluations were calculated using the Compusense software (Compusense Five data system Ver. 5.6, Canada). The significance of each descriptive attribute in discriminating between the samples was analysed using analysis of variance (ANOVA) and Tukey's honestly significant difference test (significance of differences at *p* < 0.05).

### Nutritional analyses

Total carbohydrate and free sugar composition was determined from freeze-dried samples as described (Nordlund et al. 2018). Amino acid analysis of freeze-dried samples followed a published protocol, too (Saarela et al. 2017).

### Plant growth regulator analyses

Lyophilized samples (50 mg) were homogenized with 1 ml of 80% methanol using zirconium grinding balls with a Retsch mixer mill MM400 homogenizer (20 Hz, 2 min) and subjected to ultrasonication for 20 min. The suspensions were centrifuged at 10000 rpm for 5 min. The liquid phase was transferred to another tube and the samples were re-extracted with 0.5 ml of 80% methanol. Methanol from the combined extracts was evaporated to dryness at 30 °C under a gentle stream of nitrogen. Prior to solid-phase extraction, the samples were diluted with 0.8 ml MilliQ-water.

SPE purification was performed using OASIS HLB LP 96-well Plate (60 µm, 60 mg) (Waters, pn 186000679) that was conditioned by washing first with 1.5 ml (3 × 0.5 ml) of 1% acetic acid in methanol and then with 1.5 ml (3 × 0.5 ml) of 1% acetic acid in 50% methanol and then equilibrated with 1.5 ml (3 × 0.5 ml) 1% acetic acid in 30% methanol. The diluted samples (final volume 1.0 ml) were passed thought SPE plate, followed by washing with 3 ml (3 × 1 ml) of 1 mM ammonium acetate and vacuum drying for 10 min. The analytes were eluted with 1.5 ml (3 × 0.5 ml) of 1% acetic acid in 80% ethanol. The eluted samples were evaporated to dryness at 30 °C under a gentle stream of nitrogen and reconstituted in 0.2 ml of 50% methanol.

Analyses were developed to cover six growth regulators: Kinetin (KIN, Sigma-Aldrich K0753), 1-Naphthaleneacetic acid (NAA, Sigma-Aldrich NO640), 2,4-Dichlorophenoxyacetic acid (2,4-D, Sigma-Aldrich D7299), 6-Benzylaminopurine (BA, Sigma-Aldrich B3408), 3-Indoleacetic acid (IAA, Sigma-Aldrich I2886) and Thidiazuron (TDZ, Sigma-Aldrich P6186). The analyses were performed on an Acquity UHPLC system, Waters (Milford, MA, USA) and Waters Xevo TQ-S MS (Manchester, UK). Chromatography was performed using an ACQUITY UPLC BEH HSS T3 (1.8 µm, 2.1 × 100 mm) (Waters), kept at 30 °C. The experiment was carried out at a flow rate of 0.3 ml/min with mobile phase A (5 mM ammonium acetate in water, pH 5.5) and B (5 mM ammonium acetate in methanol, pH 5.5). The gradient elution started at 30% B and increased to 80% B within 9 min, after this directly returned to initial percentage and maintained for 3 min. Mass spectrometry was carried out using electrospray ionization (ESI) in positive and negative polarity. The capillary voltage was 1.5 kV, cone voltage 30 kV, source temperature 150 °C and desolvation temperature 500 °C. The cone and desolvation gas flow were set at 150 l/h (nitrogen) and 800 l/h (nitrogen), respectively, collision gas flow rate was 0.15 ml/min.

### Acute toxicity

Acute cytotoxicity of arctic bramble and birch cell cultures was studied using freshwater crustaceans *Daphnia magna* according to DAPHTOXKIT F Magna (ISO 6341) Standard Operational Procedure (MicroBioTests Inc., Belgium). Harvested cells of birch and arctic bramble cultures were freeze-dried and extracted with 80% ethanol, which was evaporated to dryness using Rotavapor. The residue was dissolved in H_2_O and freeze-dried. For the test solutions, the freeze-dried samples were dissolved into Standard Freshwater (SF; provided with the test kit) as 1.25 mg/ml and filtered to separate non-soluble material. The filtrate was used in the test as such as highest concentration of 1.25 mg/ml, and step-wise diluted in SF into concentrations 0.25, 0.05, 0.01 mg/ml. Prior performing the test, pH values of these test solutions were measured. SF served as the positive control and K_2_Cr_2_O_7_ as the negative control for the test materials. In addition, fresh carrots (*Daucus carota*) and frozen bilberry (*Vaccinium myrtillus*) fruits bought at local supermarket were prepared similarly as described above for plant cell cultures, and their solutions were included in the analysis for comparison to human food material.

The test was performed according to the protocol included in the test kit. Briefly, dormant ephippia of *Daphnia magna* were released in SF-water and let to develop under strong illumination at 20 °C for three days. Hatched neonates were pre-fed with *Spirulina* powder for 2 hours, and the test was performed inserting five neonates per well with test solutions or controls in multiwall plate. Viability (mobility) of the neonates was recorded after 24 h and 48 h incubation at 20 °C in dark. Toxicity of the test solutions was calculated from ratio of the dead or immobilized crustaceans against total count, and indicated as EC_50_ values (effective concentration).

### Statistical analyses of biomass accumulation data

Statistical analyses were conducted with IBM SPSS Statistics 25 software. Normality of the data was assessed with Shapiro–Wilk’s test. Unless otherwise described, one-way ANOVA was used together with Tukey HSD as post-hoc test when homogeneity of variances allowed (Levene test *p* > 0.05). With data comprising unequal variances, Dunnett T3 test was used. Confidence level *p* < 0.01 was used throughout the data.

## Results and discussion

### Sucrose substitution in plant cell culture media

#### Biomass yield

Previous studies with cell cultures of *Cucumis sativus* and *Ajuga reptans* indicated that lactose-containing media can be successfully used (Callebaut and Motte 1988; Callebaut et al. 1990). In the current study, however, complete lactose-containing side streams from dairy industry were directly assessed for their suitability as medium ingredient for arctic bramble and birch cell cultures. The studied side-streams consisted of lactose fraction (LS-1), UF (ultra-filtrated) milk permeate (LS-2), acid whey (LS-3) and UF whey permeate (LS-4) (Supplementary Table S1). Lactose fractions derive from whey in milk and cheese processes following ultrafiltration to remove proteins. Both UF whey permeate and lactose fraction are high in lactose (97–120 g/l). Milk permeate is a collective term for lactose, vitamins and minerals in milk, and it is used to standardize the milk protein and fat contents to a constant level throughout the year (Hardham 1998). Acid whey is produced in acid-coagulated cheese process where lactose is converted to lactic acid. The lactose concentrations in UF milk permeate and acid whey are around 45 g/l.

**Fig. 1:**
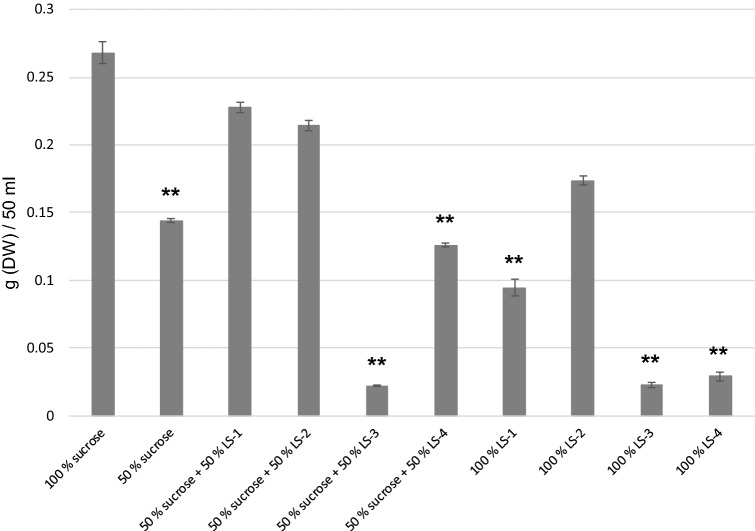
Biomass accumulation of arctic bramble cell culture in media containing different carbohydrate sources. Sucrose 100% represents 30 g/l. Asterisks (**) represent statistical differences compared to 100% sucrose with the level *p* < 0.01 (Dunnett T3)

In the case of arctic bramble cell culture, a clear potential was seen by keeping the total carbohydrate level constant at 30 g/l but substituting 50% of sucrose with either LS-1 or LS-2, resulting in a statistically non-significant decrease of biomass accumulation as compared to 100% sucrose (Fig. 1). Remarkably, complete substitution of sucrose with LS-2 did not lead to significantly reduced biomass accumulation showing the great potential of this side stream in cultivation of arctic bramble cell cultures (Fig. 1). On the other hand LS-3 and LS-4 did not perform well in replacing sucrose in cultivation of arctic bramble cell culture and LS-3 seemed to inhibit the cell culture growth (Fig. 1).

**Fig. 2:**
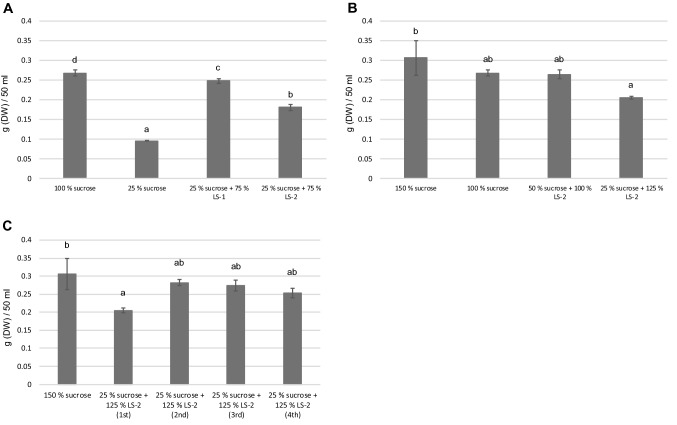
Biomass accumulation of arctic bramble cell culture in media containing different lactose-containing side-streams and/or sucrose. Sucrose 100% represents 30 g/l. Letters represent statistical differences (*p* < 0.01). **a** Sucrose replacement with lactose fraction (LS-1) and UF milk permeate (LS-2) (**b**) UF milk permeate (LS-2) ratios in media with elevated total carbohydrate levels. **c** Long-term maintenance in UF milk permeate (LS-2) containing media

To examine in more detail whether increased ratios of lactose-containing side-stream could enhance the biomass yield of plant cell cultures, both LS-1 and LS-2 were studied using amounts corresponding to 75% of the total carbohydrates in the medium (Fig. 2a). It was observed that although neither fraction with 75% of the respective side-stream could reach the biomass yield corresponding to 100% sucrose, LS-1 and LS-2 resulted in 93% and 67%, respectively, of the biomass yield compared to 100% sucrose.

Due to the promising result using LS-2 as sole carbohydrate source (Fig. 1), it was assayed whether addition of sucrose corresponding to an increased total carbohydrate concentration of 45 g/l instead of 30 g/l (150% and 100% sucrose, respectively) would lead to further dry weight gain in cultivation of arctic bramble cell culture. Indeed, medium containing 100% LS-2 with 50% sucrose resulted in the same dry weight as with 100% sucrose medium (Fig. 2b). Reducing the added sucrose further (25% sucrose with 125% LS-2) resulted in lower biomass yield, although the change being statistically non-significant when compared to 100% sucrose medium.

For the development of industrial processes, consistent behavior of plant cell culture lines during subculture cycles is critical. Maintenance in lactose-containing side-stream was assayed with the medium having 25% of the original sucrose added with 125% LS-2 for four cultivation passages of arctic bramble cell culture. Interestingly, after a clear drop in dry weight following transfer to the medium (1st passage), the dry weight stabilized over the subsequent passages at the level reached with the original sucrose medium (Fig. 2c). This is very encouraging considering long term maintenance and biomass production needs of plant cell cultures for food applications.

**Fig. 3:**
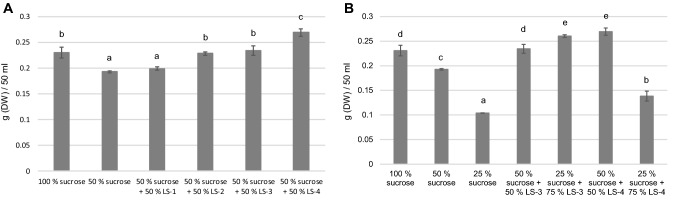
Biomass accumulation of birch cell culture in media containing different carbohydrate sources. Sucrose 100% represents 30 g/l. Letters represent statistical differences (*p* < 0.01). **a** Sucrose replacement with lactose fraction (LS-1), UF milk permeate (LS-2), acid whey (LS-3) and UF whey permeate (LS-4). **b** Replacement of sucrose with different ratios of acid whey (LS-3) and UF whey permeate (LS-4)

Optimal cell culture conditions are species and cell line specific. Therefore it was essential to evaluate the effects of sucrose replacement for a taxonomically non-related species. For this purpose above mentioned conditions involving media individually replacing sucrose with side stream fractions LS-1, LS-2, LS-3 and LS-4 were tested with birch cell culture, too. It was observed that all tested fractions resulted in equal or increased biomass generation of birch cell culture compared to 50% sucrose alone (Fig. 3a). Thus, birch cell culture responded very differently to the media as compared with arctic bramble culture. Remarkably, replacing 50% of sucrose with UF whey permeate (LS-4) resulted in statistically significantly higher biomass (17%) when compared to original medium containing only sucrose. Replacing 50% of sucrose with either LS-2 or LS-3 supported equal biomass yield as the original sucrose medium for birch cell culture.

To evaluate whether further increased amounts of lactose-containing side-stream could enhance the biomass yield of birch cell cultures, both acid whey (LS-3) and UF whey permeate (LS-4) were studied using amounts corresponding to 75% of the total carbohydrates in the medium (Fig. 3b). Interestingly, the two fractions behaved differently. UF whey permeate had a negative effect on growth when consisting 75% of the total carbohydrates, while acid whey boosted biomass yield by 13% when compared to the original medium containing only sucrose.

The two cell cultures assayed here, arctic bramble and birch cell suspension behaved differentially towards the side streams. For arctic bramble, particularly LS-2 (UF milk permeate) showed promise as replacement for sucrose. By substituting up to 83% of the amount of sucrose with lactose deriving from this side stream, 68% of the biomass yield in full sucrose medium was achieved in cultivation of arctic bramble cell culture. Moreover, the cells maintained their growth/division capacity over four cultivation passages in this medium without significant reduction in biomass. For birch cell culture, all tested side streams were successful in replacing 50% of the sucrose in the original medium. Particularly LS-3 (acid whey) showed to be promising, yielding even higher biomass yield with 75% sucrose replacement compared to 100% sucrose. It has been reported that in cucumber cell cultures, replacing part of the sucrose in the medium by lactose causes a prolonged lag-phase of the culture (Callebaut and Motte 1988). However, supplementation of the medium with low amounts of sucrose decreases the length of the lag-phase. Also, the complete inability to grow on milk whey permeate was overcome by sucrose addition to the medium. These results are partly supported by the current study of arctic bramble cell culture where good and stable growth could be seen in media with lactose-rich side streams combined with low amounts of sucrose. The enzymatic activity related to lactose metabolism corresponding to β-galactosidase was reported to increase when cells were cultivated on lactose, whereas in media with only sucrose this activity was not detected (Callebaut and Motte 1988). In the same study, the authors observed that milk whey permeate putatively contains one or more components which inhibit lactose utilization. This might be a possible explanation why in the current study we observed that UF whey permeate (LS-4) decreased the biomass of arctic bramble already with 50% sucrose replacement (Fig. 1). However, this was not seen in the case of the birch cell culture (Fig. 3). Callebaut et al. (1990) showed that callus cultures of *Ajuga reptans* were able to maintain their anthocyanin production in medium with milk whey as the sole carbon source at the same level as in the MS-based medium. This is an important observation thinking of the nutritional value and healthiness of plant cell cultures for food purposes when grown sustainably using dairy side streams as partial carbon source. However, the secondary metabolites were not studied in the current work and further experiments are needed.

#### Sensory evaluation

**Fig. 4:**
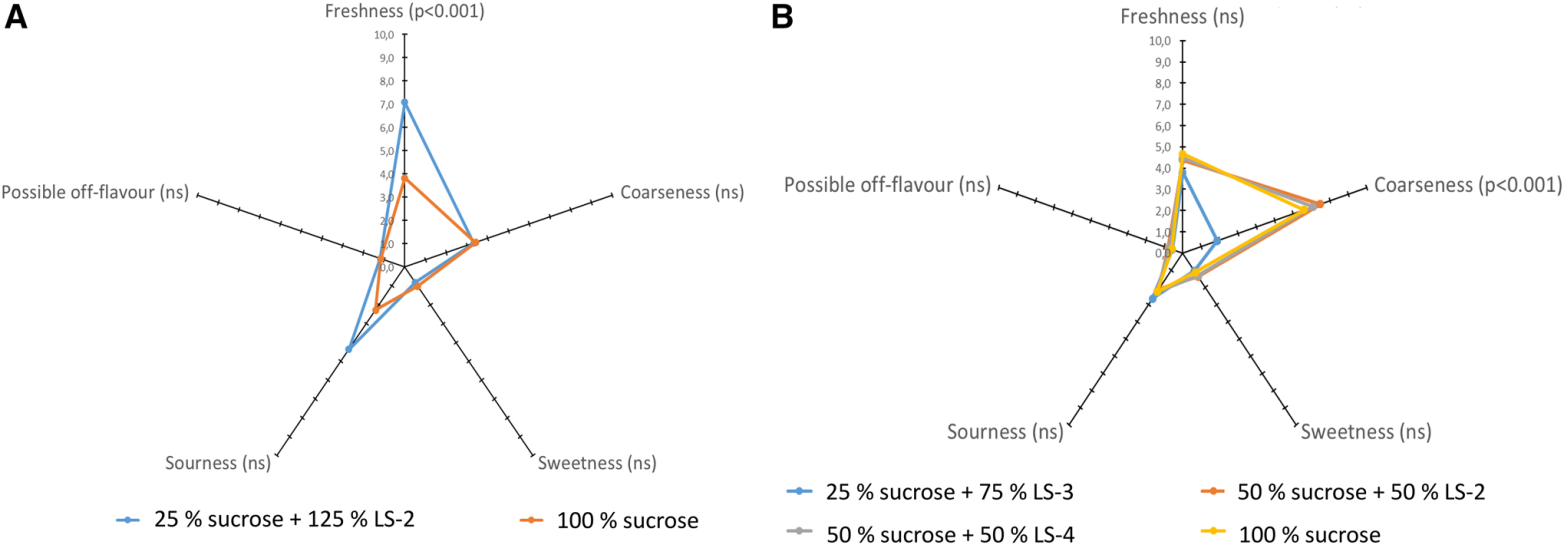
Sensory profiles of plant cell cultures of arctic bramble and birch (*n* = 10). Sucrose 100% represents 30 g/l. **a** Arctic bramble cell culture cultivated in media with 25% sucrose with 125% UF milk permeate (LS-2) or 100% sucrose. **b** Birch cell cultures cultivated in various lactose-containing side-streams or in 100% sucrose

Sensory attributes of the cell cultures cultivated either on sucrose or media where sucrose was partly replaced by lactose-containing side streams, were studied by a sensory panel with ten members. Arctic bramble cell culture cultivated in media with 100% sucrose or 25% sucrose + 125% LS-2 (with sucrose 100% representing 30 g/l) were subjected to sensory analyses to determine the effect on the sensory profile of the cultivated cell cultures. It was observed that arctic bramble cell samples differed statistically significantly from each other only in odour freshness (*p* < 0.001). The sample grown in side stream supplemented medium, i.e., with elevated total carbohydrate level scored significantly higher odour freshness than the control grown in 100% sucrose (Fig. 4a). Coarseness, sweetness, sourness and off-flavours were not significantly affected.

Birch cell culture cultivated in three different modified media showed similar or higher biomass accumulation compared to control (100% sucrose) (Fig. 3) that is why these samples were subjected to the sensory analyses. Only the culture in 25% sucrose + 75% LS-3 reduced coarse mouthfeel statistically significantly (p < 0.001) (Fig. 4B). The treatments did not otherwise show statistically significantly differences concerning the other evaluated attributes: freshness, sweetness, sourness, possible off-flavours.

Overall the sensory study showed that supplementing the culture medium with side streams can affect sensory attributes. Despite the limited number of investigated treatments, we were able to show that the sensory properties of both cell cultures, arctic bramble and birch, can be influenced by changing the carbon source. The cultivation in media with lactose side streams increased the odour freshness and decreased the coarse mouthfeel, respectively. Both changes are regarded as positive effects, i.e., improving sensory perception. The mechanisms leading to these differences remain however unclear. Flavour formation is generally very complex and plant cell cultures are under investigated in this respect. Future studies, aiming to better understand the flavour formation of plant cells, could correlate the sensory profile to selected non-volatile chemical compounds, such as acids, phenolic compounds and sugars, by e.g., statistical multivariate techniques.

#### Carbohydrate composition

Samples subjected to sensory evaluation were analysed for their carbohydrate contents to study whether alternative lactose-containing carbon sources affect intracellular carbohydrate composition. For arctic bramble, the results in Table 1 show that free sugars and total glucose were overall lower (ca. 2–5 fold less) in the sample cultivated with UF milk permeate (LS-2). Higher galactose in sample cultivated with LS-2 was observed and most probably reflects the utilisation of lactose-rich side stream and the presence of β-galactosidase as has been shown for e.g., *Cucumis sativus* and *Medicago sativa* cell cultures (Callebaut and Motte 1988; Chaubet et al. 1981). Overall, carbohydrate values were very close to the results reported by Nordlund et al. (2018) for cloudberry, lingonberry and stoneberry cell cultures grown in 100% sucrose medium.

**Table 1:** Intracellular carbohydrate composition (mg/g DW) of cell cultures of arctic bramble and birch when cultivated in medium with various carbohydrate sources

	Arctic bramble cell culture in 25% sucrose + 125% LS-2^a^	Arctic bramble cell culture in 100% sucrose^a^	Birch cell culture in 50% sucrose + 50% LS-4^b^	Birch cell culture in 25% sucrose + 75% LS-3^b^	Birch cell culture in 50% sucrose + 50% LS-2^b^	Birch cell culture in 100% sucrose^b^
Acid insoluble material [%]	3.7 (0.1)	3.0 (0.3)	9.1 (0.5)	5.5 (0.1)	9.8 (0.1)	14.9 (0.4)
Acid soluble Material [%]	6.5 (0.3)	3.8 (0.4)	3.4 (0.2)	4.9 (0.1)	4.2 (0.1)	4.3 (0.4)
Free sugars [%] (Sum of Glu, Xyl, Ara, Gal, Man, Rha, Fru, Suc)	8.4	38.3	18.4	16.9	21.0	33.3
Free sugar composition [mg/g]						
Glucose	37.4 (1.0)	164.0 (0.6)	92.4 (2.1)	79.5 (0.3)	103.0 (3.9)	173.7 (1.8)
Xylose	< 4	< 4	< 4	< 4	< 4	< 4
Arabinose	< 4	< 4	< 4	< 4	< 4	< 4
Galactose	< 4	< 4	< 4	8.2 (0.0)	< 4	< 4
Mannose	< 4	< 4	< 4	< 4	< 4	< 4
Rhamnose	< 4	< 4	< 4	< 4	< 4	< 4
Fructose	25.2 (0.9)	92.1 (2.5)	91.6 (1.5)	80.8 (1.1)	107.3 (3.1)	154.6 (1.6)
Sucrose	21.5 (1.6)	126.8 (2.1)	< 4	< 4	< 4	5.1 (0.3)
Total sugar composition [mg/g]						
Glucose^c^	146.3 (1.8)	322.3 (60.6)	171.4 (0.3)	171.9 (3.2)	184.2 (3.8)	247.3 (0.8)
Xylose	11.9 (0.2)	6.9 (0.5)	6.6 (0.2)	8.9 (0.2)	7.5 (0.3)	5.6 (0.0)
Arabinose	31.2 (0.0)	22.6 (0.0)	31.8 (1.2)	42.6 (1.4)	37.8 (0.1)	33.9 (0.2)
Galactose	61.5 (2.2)	34.4 (6.2)	39.3 (1.9)	45.1 (0.5)	44.4 (0.7)	27.7 (0.2)
Mannose	3.9 (0.1)	3.7 (0.2)	6.5 (0.1)	5.8 (0.1)	7.3 (0.1)	6.9 (0.0)
Rhamnose	8.6 (0.1)	5.0 (0.6)	6.2 (0.1)	8.0 (0.1)	6.8 (0.1)	4.4 (0.0)
Fructose^d^	nd	nd	nd	nd	nd	nd

Results are expressed as mean (± SD) of two biological replicates

^a^45 g/l total amount of carbohydrates

^b^30 g/l total amount of carbohydrates

^c^Contains also glucose from sucrose (sucrose degraded in the acid hydrolysis used in the analysis)

^d^Fructose was not detectable by the method used

For birch, similarly as for arctic bramble, free sugars and total glucose were overall lower (ca. 1.5–2 fold less) in the side-stream cultivated samples (Table 1). Again, higher galactose levels in the sample cultivated with LS-2 were observed most probably reflecting direct uptake from the culture medium after the hydrolysis of lactose to galactose and glucose moieties.

#### Amino acid composition

The samples which were analysed for their carbohydrates were analysed for their amino acid contents, too. Results are shown in Table 2. In the case of arctic bramble, all amino acids besides arginine were higher in the sample cultivated in LS-2 supplemented medium. The total amount of amino acids in this sample was also significantly higher, around 1.5-fold compared to the control. It is interesting to note that this sample also scored increased odour freshness in the sensory evaluation. However, it is not known whether these differences in amino acid levels could contribute to the odour freshness.

**Table 2:** Amino acid concentrations in arctic bramble and birch cell culture samples after 8-day cultivation in control (100% sucrose) and in modified media

Amino acid content [mg/g] dw	Arctic bramble cell culture in 25% sucrose + 125% LS-2^a^	Arctic bramble cell culture in 100% sucrose^a^	Birch cell culture in 50% sucrose + 50% LS-4^b^	Birch cell culture in 25% sucrose + 75% LS-3^b^	Birch cell culture in 50% sucrose + 50% LS-2^b^	Birch cell culture in 100% sucrose^b^
His	6.8 ± 0.2	4.7 ± 0.1	3.8 ± 0.1	3.3 ± 0.2	3.4 ± 0.0	3.5 ± 0.1
Ser	15.3 ± 0.4	10.8 ± 0.4	9.0 ± 0.5	10.5 ± 0.5	9.6 ± 0.1	10.8 ± 0.3
Arg	12.7 ± 0.2	16.0 ± 0.2	7.0 ± 0.3	6.0 ± 0.3	6.9 ± 0.3	6.6 ± 0.4
Gly	10.3 ± 0.3	7.0 ± 0.1	6.2 ± 0.2	5.2 ± 0.2	5.6 ± 0.1	5.7 ± 0.1
Asp	31.3 ± 0.2	15.8 ± 0.3	14.5 ± 0.3	13.2 ± 0.6	13.6 ± 0.1	13.2 ± 0.2
Glu	39.6 ± 0.2	21.2 ± 0.4	17.1 ± 0.4	15.7 ± 0.8	17.3 ± 0.2	16.1 ± 0.2
Thr	10.3 ± 0.3	6.6 ± 0.1	6.1 ± 0.2	5.7 ± 0.3	5.5 ± 0.0	5.6 ± 0.2
Ala	14.2 ± 0.2	10.7 ± 0.2	7.8 ± 0.2	6.7 ± 0.4	6.8 ± 0.0	6.7 ± 0.1
Pro	9.1 ± 0.2	5.9 ± 0.2	5.9 ± 0.2	5.6 ± 0.2	5.4 ± 0.0	5.5 ± 0.1
Cys	1.8 ± 0.1	1.2 ± 0.1	1.6 ± 0.1	1.5 ± 0.1	1.4 ± 0.1	1.1 ± 0.1
Lys	14.3 ± 0.5	9.9 ± 0.3	9.3 ± 0.2	7.3 ± 0.4	8.5 ± 0.1	7.8 ± 0.1
Tyr	6.3 ± 0.2	5.0 ± 0.2	1.3 ± 0.1	2.8 ± 0.2	3.5 ± 0.0	3.3 ± 0.0
Met	4.6 ± 0.1	3.1 ± 0.1	3.1 ± 0.1	2.7 ± 0.1	2.8 ± 0.0	3.0 ± 0.0
Val	14.9 ± 0.1	9.8 ± 0.2	8.2 ± 0.4	7.5 ± 0.5	7.7 ± 0.2	7.6 ± 0.2
Ile	9.8 ± 0.2	7.1 ± 0.2	6.8 ± 0.3	6.5 ± 0.4	6.3 ± 0.0	6.2 ± 0.1
Leu	16.6 ± 0.1	11.4 ± 0.2	11.7 ± 0.3	10.7 ± 0.5	10.5 ± 0.1	10.8 ± 0.1
Phe	9.9 ± 0.4	6.5 ± 0.2	7.1 ± 0.3	6.5 ± 0.4	6.1 ± 0.1	6.3 ± 0.1
Trp	1.4 ± 0.0	1.2 ± 0.0	0.8 ± 0.0	0.7 ± 0.0	0.9 ± 0.0	0.8 ± 0.0
SUM as %	229.1 ± 3.0	154.1 ± 2.6	127.4 ± 3.7	118.1 ± 5.8	121.9 ± 0.6	120.5 ± 2.1

The results are expressed as mg/g dw ± SD (*n* = 3). The calculated sum of the amino acids is considered to present the total protein content of the sample

^a^45 g/l total amount of carbohydrates

^b^30 g/l total amount of carbohydrates

In the case of birch cell cultures, no major differences were observed when samples grown in media with lactose side streams were compared to the control grown in basic culture medium with 100% sucrose.

### Growth regulators in plant cell cultures

#### Plant growth regulator accumulation

Synthetic growth regulators are used as pesticides in agriculture and their use is regulated by law in Europe (https://ec.europa.eu/food/plant/pesticides/eu-pesticides-database/public/?event=homepage&language=EN). In this study, we used KIN, NAA and 2,4-D as growth regulators in the cultivation arctic bramble and birch cell cultures. For NAA the acceptable daily intake (ADI) is 0.1 mg/kg body weight/d and the acute reference dose (ARfD) is 0.1 mg/kg body weight. For 2,4-D, the ADI is 0.02 mg/kg body weight/d and the ARfD is 0.3 mg/kg body weight. For both compounds there are additionally maximum residue levels (MRL) in various crops defined, e.g., in blueberries 0.06 mg/kg and 0.1 mg/kg, respectively. KIN is not listed as a pesticide in the EU database. The ADI, ARfD and acceptable operator exposure levels (AOEL) values for all assayed growth regulators can be found in Supplementary Table S2. Here, we monitored concentrations of growth regulators after one growth passage in light and dark conditions. Targeted UPLC-MS/MS was employed for the detection of analytes based on their mass transitions: 226.2 > 91.1 for BA, 176.1 > 130.1 for IAA, 216.1 > 81.1 for KIN, 204.2 > 141.1 for NAA, 221.1 > 102.1 for TDZ and 219.0 > 161.0 for 2,4-D. Original arctic bramble medium contains 0.1 mg/l KIN and 1.0 mg/l NAA, and birch medium 2.0 mg/l 2,4-D and 0.5 mg/l KIN (Table 3). Only 2,4-D levels were above detection limit in the birch cultures cultivated for 8 days (harvesting point) (Table 3). The amounts of residual 2,4-D in light cultivation of birch cell culture was higher as compared to dark cultures both in the cells and in the medium. Previous auxin starvation studies in plant cell cultures have revealed that both partial and complete auxin starvation results in substantially increased accumulation of the endogenous free indole-3-acetic acid content in exponential growth phase (Zažimalová et al. 1995). The metabolism of 2,4-D in plant cells has been studied by labelled 2,4-D feeding with wheat cell suspensions (Bristol et al. 1977) and although not quantified, it has been suggested that main metabolite routes for 2,4-D in plants proceed via detoxification by ring hydroxylation, and amino acid conjugation. Especially herbicide resistant monocotyledonous species exhibit formation of carboxylic glycosides of 2,4-D, too. Scheel and Sandermann (1981) reported that 2,4-D and its metabolites were conjugated with lignin and deposited in cell wall structures in wheat and soybean cell suspension cultures. Thus, it should be noted that 2,4-D commonly forms various types of conjugates in living cells. The fact that in this study only minute amounts of 2,4-D were found as free form during harvesting does not necessary mean that 2,4-D toxicity can be ruled out. For this reason, these cell cultures were subjected in this study to a toxicity assessment using living freshwater crustaceans.

**Table 3:** Concentrations of plant growth regulators used in the original medium and after 8-day cultivation in either light or in darkness in the intracellular space of arctic bramble and birch cell cultures (μg/g DW; ± SD; *n* = 3)

Cell culture	Growth regulator	Culture medium	Intracellular (8 days) light	Intracellular (8 days) dark
		mg/l	μg/g DW	μg/g DW
Arctic bramble	BA	0	ND	ND
	IAA	0	ND	ND
	KIN	0.1	ND	ND
	NAA	1.0	ND	ND
	TDZ	0	ND	ND
	2,4-D	0	ND	ND
Birch	BA	0	ND	ND
	IAA	0	ND	ND
	KIN	0.5	ND	ND
	NAA	0	ND	ND
	TDZ	0	ND	ND
	2,4-D	2.0	0.82 ± 0.23^a^	0.33 ± 0.05^b^

*ND* not detected, below the limit of quantitation (LOQ: 0.1 pg/l for KIN, 50.0 pg/l for NAA, 0.4 pg/l for 2,4-D, 0.1 pg/l for BA, 0.1 pg/l for IAA, 0.1 pg/l for TDZ)

^a^If calculated from the intracellular concentration when taking into consideration, the biomass (DW) produced per litre, the corresponding amount was 0.016 ± 0.004 mg/l

^b^If calculated from the intracellular concentration when taking into consideration the biomass (DW) produced per litre, the corresponding amount was 0.001 ± 0.000 mg/l

#### Acute toxicity of plant cells

Freshwater crustaceans (*Daphnia magna*) were used to evaluate the acute toxicity of the studied plant cell cultures (Table 4). To evaluate the toxicity, the EC_50_ value was calculated, representing the concentration of the test compound in which 50% of *Daphnia* neonates die and/or are immobilized during 24 and 48 h of incubation. Thus, increasing toxicity of the compound is reflected by a decreasing EC_50_ value. EC_50_ values of the birch and arctic bramble cell culture extracts were 760 and 730 mg/l, respectively, after 48 h incubation indicating very weak toxic effect against *Daphnia magna*, when compared to 0.6 mg/l of K_2_Cr_2_O_7_ used as control for toxicity. The difference in EC_50_ values of birch and arctic bramble cell extracts was considerable after 24 h incubation, but indicated very little toxicity for birch and not detectable for arctic bramble. For comparison, extract prepared from carrots (*Daucus carota*) was somewhat least toxic, whereas bilberry (*Vaccinium myrtillus*) fruits showed the lowest EC_50_ values indicating highest toxicity of the samples tested.

**Table 4:** Acute toxicity assessment of plant cell cultures and edible plant samples with *Daphnia magna*

	EC_50_ values (mg/L)	
Measured values	24 h	48 h
Arctic bramble cell extract	> 1250	730
Birch cell extract	820	760
Carrot extract	> 1250	1000
Bilberry fruit extract	730	730
K_2_Cr_2_O_7_ (negative control)	1	0.6
Reference values according to literature	24 h	48 h
2,4-D^a^	~ 300	nd
Malathion^a^	~ 0.004	nd
Na-PCP^a^	~ 1	nd
K2Cr2O7^a^	~ 1	nd
Ethanol^b^	nd	~ 10
Streptomycin^c^	947	487
NAA^d^	nd	180

Results presented as EC_50_ values after 24 h and 48 h

*nd* not determined

^a^Persoone et al. (2009) . In: Knowledge and Management of Aquatic Ecosystems, 393, 01

^b^Barbosa et al. 2003. Bull. Environ. Contam. Toxicol. 70

^c^Wollenberger et al. 2000. Chemosphere 40

^d^https://ecotox.ipmcenters.org/details.cfm?recordID=8254

The challenge in the *Daphnia magna* acute toxicity test is the sensitivity of this organism to various physical and chemical factors, such as non-dissolved particles, dark-colored liquids, low pH and various natural compounds, such as organic acids (Pintar et al. 2004; Persoone et al. 2009; El-Deeb Ghazy et al. 2011). Acidic environment below pH 5.5 is detrimental to *Daphnia magna* (Chen et al. 2012), and the very low pH of pH 4.1 of bilberry fruit extract at the highest concentration (1.25 mg/ml) most potentially caused the strong toxic effect detected. The birch cell solution of 1.25 mg/ml was acidic with pH 6.0, whereas arctic bramble and carrot solutions were neutral, which is still below the optimum conditions from pH 7.9 to pH 8.3 detected for *Daphnia magna* (Ghazy et al. 2011).

Arctic bramble (*Rubus arcticus*) is a Nordic plant with edible berries containing delicious taste and very nice flavour, but the annual crop of wild berries is rather low and cultivated plants do not flourish well. Silver birch (*Betula pendula*) is a common forest and park tree located especially in Nordic temperate zones. The wooden part is used for timber and firewood, sap for drinks and xylitol, and leaves for tea. To our best knowledge, there are no reports of toxic effects of these plants. However, purified compounds as well as metabolites originating from these plants, such as organic acids may cause toxic effects (Pintar et al. 2004). In addition, low pH during incubation is also shown to modify compounds into more toxic against *Daphnia magna* (Anskjær et al. 2013). Chemical composition of plant cell cultures may differ significantly from that of the original plant, and cultivation parameters and medium components increase variations in their chemical composition. In Table 4, the reference values of EC_50_ concentrations of six different compounds are presented, according to the literature. Both arctic bramble and birch cell cultures showed EC_50_ values closest to the toxicity of streptomycin. When it comes to toxicity as oral doses, the oral LD_50_ of 2,4-D in the rats, mice and guinea pig ranges from 320 to 1000 µg/g. Thus the amount of free 2,4-D found in the harvesting point in a portion of 100 g DW birch cell culture would clearly stay below reported LD_50_ levels.

#### Replacement of plant growth regulators

Caseine, yeast extract, coconut water and IAA were studied for their usefulness as substitutes of growth regulators kinetin (KIN) and potentially problematic 1-naphtaleneacetic acid (NAA) in arctic bramble cultures. In addition, the possible effect of light on biomass accumulation was studied.

**Fig. 5:**
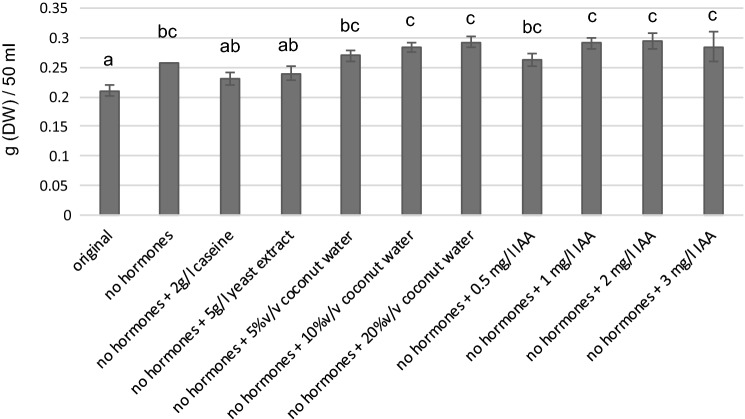
Arctic bramble cell culture cultivated with and without growth regulators and with various culture medium additives: caseine, yeast extract, coconut water, IAA. Cultivation period was 8 days. Letters represent statistical differences (*p* < 0.01)

**Fig. 6:**
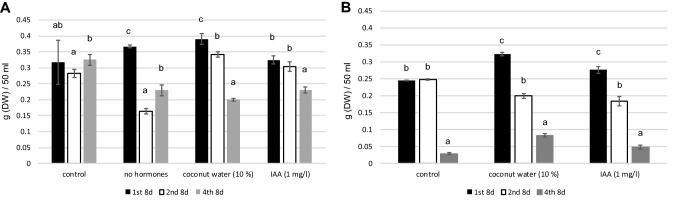
Arctic bramble cell culture cultivation over four cultivation passages in original culture medium with NAA + KIN, medium without hormones, medium without hormones + 10% coconut water and in medium without hormones + 1 mg/l IAA. Black: 1st cultivation passage; White: 2nd cultivation passage; Grey: 4th cultivation passage. Letters represent statistical differences in each case as a function of cultivation passage (*p* < 0.01). **a** in light and (**b**) in darkness

When growth regulators were omitted, the biomass yield of arctic bramble cell culture increased after the first passage (Fig. 5). However, this effect was temporary and the growth of cell cultures decline over time without growth regulators (Fig. 6). Caseine (2 g/l) and yeast extract (5 g/l), added to compensate the omitted growth regulators, did not result in increased biomass compared to original or medium without growth regulators (Fig. 5). Coconut water, when added at concentrations of 5, 10 and 20% (v/v), resulted in slightly increased biomass (up to 14%). A similar effect was observed with the addition of 1 mg/l IAA. For further experiments, coconut water 10% (v/v) and IAA (1 mg/l) were selected.

Light is an important physical factor in some plant cell cultures. However, growing illuminated cultures requires more technical efforts and energy and is therefore more costly. Therefore it was assessed whether arctic bramble cell culture could be cultivated in dark conditions. Omission of hormones led to a significant decrease in biomass accumulation after the second subculture in light and improved only slightly after the fourth subculture (Fig. 6a). In dark conditions, the dry weight of the control culture remained constant after the second subculture in darkness but dropped extremely after the fourth passage (Fig. 6b). A combination of darkness and hormone omission was therefore not followed up.

In the early days of plant cell cultures coconut water and casein hydrolysate were widely used to support cell growth. Several reports postulate the reason for growth promoting effects of coconut water, casein hydrolysate and yeast extract. For example, in coconut water, 75% of the free amino acids consist of glutamine, alanine and aminobutyric acid (Carew and Staba 1965). Furthermore, coconut water exhibits particular growth stimulating activities, deriving from reduced nitrogen, myo-inositol and sorbitol, as well as uncharacterized substances promoting cell division (Steward et al. 1961). In medium supplemented with 10% coconut water or 1 mg/l IAA, biomass of arctic bramble cell culture can be maintained at the same level as the control over two passages (16 d) in light (Fig. 6a) and only for one passage (8 days) in darkness (Fig. 6b). This is a promising result when thinking of the cultivation of plant cell cultures for food purposes. Further studies are needed to analyse techno-economics and life cycle of the plant cell cultures for food-concept.

## Conclusions

Plant cell culture technology is a viable option for production of various added value substances, especially when containment is required. For decades plant cell cultures have been exploited for generation of e.g., high-value pharmaceuticals, the noteworthy commercial successes displayed by paclitaxel and docetaxel production via Plant Cell Fermentation (PCF®) Technology by Phyton Biotech and taliglucerase alfa by Protalix Biotherapeutics. Recently, the possibilities for the usage of plant cell cultures has been broadened towards food use and we have demonstrated that undifferentiated plant cells for food are promising for their good nutritional value. Although this technology is unlikely to compete food derived from agriculture, the concept of plant cell cultures as food constitutes an additional tool for global food production and possibly to nutraceutical industry, and opens possibilities in places where traditional agriculture is in constraint, e.g., due to climate or space. However, there are still aspects of this technology which need to be studied before commercial products can be launched, including safety and flavour aspects as investigated here with arctic bramble and birch cell suspension cultures. Ultimately, food regulation compliance must be met, e.g., novel food regulation in Europe.

In this study, we showed that different lactose containing dairy side streams can be applied to replace even the majority of the normally used sucrose in plant cell culture medium, with a clear benefit for biomass accumulation. Furthermore, we also showed that the beneficial nutritional composition and sensory properties are not compromised when applying these lactose-rich dairy side streams. Dairy industry side streams can improve flavour properties of plant cell cultures but can be applied only for temporary cultivation. Growth regulators kinetin and 1-naphthaleneacetic acid can be temporarily replaced with coconut water or indole-3-acetic acid. This approach can be envisaged in two-stage cultivation systems, where maintenance of the cells is performed in original medium, while scaling up in the larger cultivation volumes or for the production phase, cells can be transferred to plant growth regulator-free medium. This was shown to be feasible also in dark conditions for arctic bramble, which is a clear advantage when considering current bioreactor designs, where lighting poses major challenges. However, it should be noted that although the biomass can be maintained for one cultivation passage in the dark, the nutritional properties (e.g., vitamins, phenolic compounds, carotenoids) might change and therefore composition should be studied before selecting dark cultivation.

On the basis of EC_50_ values obtained in the *Daphnia magna* toxicity test reported here, arctic bramble and birch cell culture extracts did not show considerable toxicity and toxicity did not exceed that of edible bilberry fruit. To our best knowledge, there is no earlier report of using *Daphnia magna* for the toxicity evaluation of plant cells. For edibility assessment of the cell cultures, more thorough toxicity evaluation is needed, as also required by the novel food legislation.

## Electronic supplementary material

Below is the link to the electronic supplementary material.

Additional file1 (PDF 33 kb)

Additional file2 (DOCX 22 kb)

Additional file3 (DOCX 22 kb)

## Data Availability

The datasets generated during and/or analysed during the current study are available from the corresponding author on reasonable request.
