# Supplementary material for: Plant cell cultures as food—aspects of sustainability and safety
Source: Plant Cell Rep. 2020 Sep 6;39(12):1655–68. doi: 10.1007/s00299-020-02592-2 (PMC7644541; doi:10.1007/s00299-020-02592-2)
Supplement: Supplementary file 1 — Additional file1 (PDF 33 kb) [file 299_2020_2592_MOESM1_ESM.pdf]

VTT

RE: Request for ethical assessment of sensory evaluation at VTT/Raija-Liisa Heiniö

The planned sensory evaluation will be conducted according to ethical standards of EU, does not include novel foods as regulated in the new Regulation (EU) 2015/2283, follows the EU General Data Protection Regulation (EU) 2016/679 and takes in consideration Occupational safety by following GRI (Global Reporting Initiative). The protocol for performing the sensory evaluation has been accepted as applied by the Ethical Committee of VTT.

On behalf of the Ethical Committee of VTT,

Richard Fagerström

Members of the Ethical committee of VTT:

Richard Fagerström, Principal Scientist
Matti Karhunen, Vice President, General Counsel
Jarmo Siivinen, Research Scientist
Seppo Viinikainen, Compliance Officer
