# Supplementary material for: Plant cell cultures as food—aspects of sustainability and safety
Source: Plant Cell Rep. 2020 Sep 6;39(12):1655–68. doi: 10.1007/s00299-020-02592-2 (PMC7644541; doi:10.1007/s00299-020-02592-2)
Supplement: Supplementary file 2 — Additional file2 (DOCX 22 kb) [file 299_2020_2592_MOESM2_ESM.docx]

**Supplementary Table S1.** Composition of lactose-containing side-streams (% w/w) and pH.

|  | Lactose fraction  LS-1 | UF-milk permeate  LS-2 | Acid whey  LS-3 | UF-whey permeate  LS-4 |
| --- | --- | --- | --- | --- |
| Dry matter | 14.6 | 5.6 | 5.9 | 12.0 |
| Protein | 0.10 | 0.00 | 0.12 | 0.03 |
| Nitrogen (non-protein) | 0.00 | 0.03 | 0.05 | 0.04 |
| Carbohydrate | 12.0 % lactose  = 120 g/l | 4.6 % lactose  = 46 g/l | 4.5 i.e. 0.9 % lactose;  1.8 % galactose;  1.8 % glucose  = 9 g/l lactose  total sugar content 45 g/l | 9.7 % lactose  = 97 g/l |
| Lactic acid | 0.03 | 0.15 | 1.00 | 0.17 |
| Citric acid | >0.02 | <0.02 | <0.02 | 0.10 |
| Ash | 0.04 | 0.50 | 0.80 | 0.68 |
| pH | 6.7 | 6.7 | 4.6 | n. 6 |
