# Supplementary material for: Plant cell cultures as food—aspects of sustainability and safety
Source: Plant Cell Rep. 2020 Sep 6;39(12):1655–68. doi: 10.1007/s00299-020-02592-2 (PMC7644541; doi:10.1007/s00299-020-02592-2)
Supplement: Supplementary file 3 — Additional file3 (DOCX 22 kb) [file 299_2020_2592_MOESM3_ESM.docx]

**Supplementary Table S2.** Acceptable daily intake (ADI), acute reference dose (ARfD) and acceptable operator exposure levels (AOEL) of assayed growth regulators according to EU Pesticides database (<https://ec.europa.eu/food/plant/pesticides/eu-pesticides-database/public/?event=homepage&language=EN>).

| **Growth regulator** | **Acceptable daily intake (ADI)** | **Acute reference dose (ARfD)** | **Acceptable operator exposure levels (AOEL)** |
| --- | --- | --- | --- |
|  | **mg/kg body weight/d** | **mg/kg body weight** | **mg/kg body weight/d** |
| **BA** | Not listed | | |
| **IAA** | Not listed | | |
| **KIN** | Not listed | | |
| **NAA^a^** | 0.1 | 0.1 | 0.07 |
| **TDZ^b^** | Not approved, no classification, no toxicological information | | |
| **2,4-D^c^** | 0.02 | 0.3 | 0.02 |

^a^ Default maximum residue levels (MRL) of 0.06 mg/kg in e.g. strawberries, blueberries, currants, cranberries and gooseberries according to Reg 396 / 2005

^b^ Default MRL of 0.01 mg/kg according to Reg 396 / 2005

^c^ Default MRL of 0.1 mg/kg in e.g. strawberries, blueberries, currants, cranberries and gooseberries according to Reg 396 / 2005
